# Genetic insights into alcohol-associated liver disease: integrative transcriptome-wide analysis identifies novel susceptibility genes

**DOI:** 10.3389/fmed.2025.1623367

**Published:** 2025-07-31

**Authors:** Qianchang Wang, Zhe Wang, Minzhe Hu, Fangfeng Liu, Zhengjian Wang

**Affiliations:** ^1^Graduate School, Shandong First Medical University, Jinan, Shandong, China; ^2^Shandong Provincial Hospital Affiliated to Shandong First Medical University, Jinan, Shandong, China

**Keywords:** alcohol- associated liver disease (ALD), genome-wide association study (GWAS), transcriptome-wide association study (TWAS), multi-marker analysis of GenoMic annotation (MAGMA), Mendelian randomization (MR), drug enrichment analysis

## Abstract

**Background:**

Alcohol-associated liver disease (ALD) is a chronic condition influenced by both genetic and environmental factors. While GWAS has identified ALD-related loci (PNPLA3, MBOAT7, TM6SF2), underlying genetic mechanisms and therapeutic targets remain unclear.

**Methods:**

This study utilized the FinnGen R12 dataset (488,982 participants) and GTEx v8 eQTL data to perform a cross-tissue transcriptome-wide association study (TWAS) using UTMOST, with single-tissue validation via FUSION. Gene-level association analysis (MAGMA), Mendelian randomization (MR), and colocalization were applied to evaluate causal links. Functional significance was assessed through GeneMANIA, drug enrichment, and molecular docking analyses.

**Results:**

Three ALD susceptibility genes—AFF1, C4orf36, and HSD17B13—were identified and validated. HSD17B13 may reduce ALD risk via lipid metabolism and redox balance, while AFF1 is implicated in transcription regulation. C4orf36 requires further study. Drug enrichment analysis highlighted AFF1 as a target for beta-D-allopyranose, dexbrompheniramine, and (+)-chelidonine, with molecular docking confirming strong binding potential.

**Conclusion:**

This study identifies AFF1, C4orf36, and HSD17B13 as ALD susceptibility genes, proposing AFF1 as a potential therapeutic target, paving the way for precision medicine in ALD, though further experimental validation is needed to establish their functional relevance.

## Introduction

Alcohol-associated liver disease (ALD) is a chronic liver condition resulting from prolonged excessive alcohol intake. Its spectrum includes alcoholic fatty liver (AFL), alcoholic hepatitis (AH), and alcoholic cirrhosis (AC), which may ultimately progress to end-stage liver disease (ESLD) or hepatocellular carcinoma (HCC) (1). With rising global alcohol consumption, the incidence and mortality of ALD have steadily increased, significantly contributing to the global liver disease burden (2, 3).

Although prolonged excessive alcohol consumption is the primary trigger of ALD, susceptibility to ALD varies significantly among individuals. Even with similar levels of alcohol intake, some individuals may never develop severe liver damage, while others may progress rapidly to ESLD (4). In recent years, genome-wide association studies (GWAS) have progressively identified multiple genetic risk loci associated with ALD, providing crucial insights into its pathogenic mechanisms. Buch et al. (5) confirmed the pivotal role of PNPLA3 (patatin-like phospholipase domain-containing protein 3), which regulates hepatic lipid metabolism by modulating triglyceride hydrolysis and lipid droplet remodeling, in ALD progression. In the same study, MBOAT7 and TM6SF2 were identified as novel genetic risk factors for ALD, both of which also participate in lipid remodeling pathways within hepatocytes (5). Subsequent GWAS have further expanded the list of ALD susceptibility genes. For instance, Innes et al. (6), Gastroenterology) reported that MARC1 exerts a protective effect (OR ∼0.76), while HNRNPUL1 variants increase ALD risk (OR ∼1.3) in heavy drinkers from the UK Biobank cohort (7). Moreover, HSD17B13 is a liver-enriched lipid droplet-associated enzyme that plays a protective role in hepatic homeostasis. It is thought to exert its hepatoprotective effects by modulating retinol and lipid metabolism, thereby attenuating hepatic inflammation and fibrogenesis. Abul-Husn et al. (7), NEJM demonstrated that these mutations lower ALD-related inflammation and fibrosis, thereby reducing liver disease progression risk by 30%–50% (6). Schwantes-An et al. (8), Hepatology integrated multiple GWAS datasets and validated the key roles of PNPLA3 and HSD17B13 in ALD, while identifying FAF2 variants as potentially protective (OR ∼0.61) (9). Notably, genetic susceptibility to ALD may differ across populations. Kim et al. (10), Hepatology conducted a GWAS in a Korean cohort and identified HNF1A as a novel genetic risk locus for ALD, with its effects potentially modulated by individual alcohol consumption levels (10).

Although GWAS has identified multiple genetic risk factors for ALD, its genetic landscape remains intricate, shaped by polygenic influences and environmental interactions. Notably, traditional GWAS are limited in their ability to pinpoint causal genes, as many associated variants are located in non-coding regions and their functional impact remains unclear.

To overcome these challenges, transcriptome-wide association studies (TWAS) integrate GWAS summary statistics with expression quantitative trait loci (eQTL) data, thereby enabling the identification of gene-trait associations with greater biological relevance and mechanistic insight. Compared to single-tissue TWAS, cross-tissue TWAS (UTMOST) improves gene prediction reliability by incorporating multi-tissue data (11). This method utilizes a “group-lasso penalty” mechanism, preserving tissue-specific eQTL effects while detecting shared effects across tissues, thereby enhancing gene expression prediction accuracy (12). In recent years, cross-tissue TWAS has been widely used to identify susceptibility genes for complex diseases (13–17), highlighting the potential involvement of tissues beyond the liver in ALD pathogenesis.

In this study, we utilized large-scale ALD GWAS data and integrated GTEx v8 eQTL datasets to identify potential susceptibility genes using cross-tissue TWAS (UTMOST). The findings were further validated at the single-tissue level using the FUSION method. Furthermore, we conducted gene enrichment analysis with MAGMA and utilized Mendelian randomization (MR) and colocalization analysis to assess causal links between candidate genes and ALD. Finally, to explore potential therapeutic targets for ALD, we performed drug enrichment analysis and molecular docking studies to elucidate the pathogenic mechanisms and therapeutic implications of the identified susceptibility genes.

## Materials and methods

**FIGURE 1:**
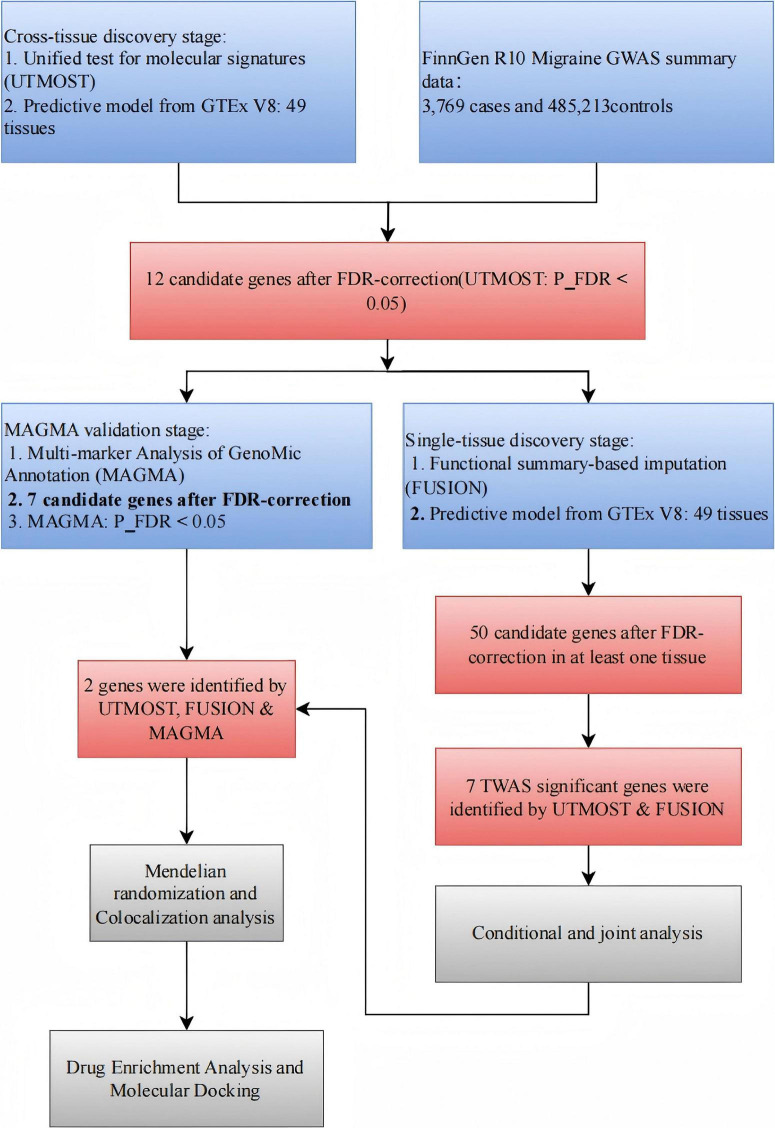
The flowchart of this study. GWAS, genome-wide association; GTEx, Genotype-Tissues Expression Project; TWAS, transcriptome-wide association studies; UTMOST, unified test for molecular signatures; FUSION, functional summary-based imputation; MAGMA, multi-marker analysis of GenoMic annotation.

The analytical workflow is depicted in Figure 1.

### ALD GWAS data source

This study utilized the FinnGen R12 GWAS dataset^1^ (18), including 3,769 ALD cases and 485,213 controls of European ancestry. Genotyping was performed using Illumina and Affymetrix arrays, followed by stringent quality control to exclude individuals with discordant sex, high missingness (> 5%), or excess heterozygosity, and variants with high missingness (> 2%), Hardy-Weinberg equilibrium *p* < 1 × 106, or minor allele count < 3. Imputation was conducted with Beagle 4.1 using the SISu v3 reference panel. Genome-wide association analysis was carried out with SAIGE (v0.35.8.8), a mixed-model logistic regression method designed to control for type I error and relatedness in large-scale, imbalanced cohorts. The association model included sex, age, batch, and the top ten principal components as covariates. All analyses were performed under a case-control design to identify SNPs associated with ALD susceptibility.

### eQTL data source

The expression quantitative trait loci (eQTL) data utilized in this study were sourced from the GTEx V8 dataset (19) which includes RNA sequencing data from 49 tissues collected from 838 post-mortem donors. Tissue sample sizes varied, ranging from 73 in the renal cortex to 706 in skeletal muscle. This study primarily concentrated on liver, gastrointestinal, and adipose tissues to enhance the characterization of gene expression patterns associated with ALD.

### Cross-tissue transcriptome-wide association study (TWAS)

Cross-tissue TWAS was performed using UTMOST (v2.0^2^), integrating GWAS summary statistics with GTEx V8 multi-tissue eQTL data via a penalized regression framework. UTMOST applies both lasso (λ_1_) and group-lasso (λ_2_) penalties to jointly model tissue-specific and shared eQTL effects, with λ_1_ and λ_2_ hyperparameters optimized by 5-fold cross-validation (group-lasso penalty λ_2_ is set to 0.5 by default, as recommended in the original UTMOST implementation) (12, 13). A ± 1Mb cis-window was used around each gene. Gene-trait associations were assessed using the generalized Berk-Jones (GBJ) test (20), and statistical significance was defined at FDR < 0.05 (Benjamini-Hochberg correction).

### Single-tissue TWAS analysis

Single-tissue TWAS was conducted using FUSION^3^, integrating ALD GWAS data with eQTL weights from 49 GTEx V8 tissues (19). FUSION evaluates five predictive models (BLUP, BSLMM, LASSO, Elastic Net, and top1), automatically selecting the model with the highest cross-validated R^2^ for association testing (21). The cis-window for SNP selection was ± 500 kb around each gene, and the LD reference was the 1,000 Genomes Project European panel (phase 3). LD clumping was performed using the default threshold of r^2^ < 0.001 within a 500 kb window. Candidate genes were defined as those reaching FDR < 0.05 in both cross-tissue and at least one single-tissue analysis.

### Conditional and joint analysis

In transcriptome-wide association studies, multiple associated features may be identified within a locus, and it is essential to distinguish which signals are truly independent. Therefore, we performed conditional and joint (COJO) analysis using the post-process module in the FUSION framework to account for linkage disequilibrium (LD) and to identify conditionally independent gene-trait associations (22, 23). In this approach, all genome-wide significant SNPs within a defined locus window (± 500 kb around the gene) were jointly modeled, conditioning on the top associated variant to estimate their independent effects while leveraging the 1,000 Genomes European reference panel to accurately capture LD patterns. This method minimizes spurious associations arising from correlated variants and provides a more comprehensive understanding of the genetic architecture underlying trait variation.

### Gene-based association analysis

Gene-based association analysis was performed using MAGMA (v1.08^4^) with all parameters set to default (24), including the SNP-wise mean model and no window extension to gene boundaries unless specified by the annotation file (25, 26). The LD reference was the 1,000 Genomes phase 3 European panel, and statistical significance was defined at FDR < 0.05 (24).

### Mendelian randomization and Bayesian colocalization analysis

We performed Mendelian randomization (MR) analysis using the “TwoSampleMR” R package (27). In this framework, cis-eQTL SNPs served as instrumental variables (IVs), gene expression as the exposure, and ALD GWAS results as the outcome. Genome-wide significant SNPs (*p* < 5E-08) were first identified, followed by LD clumping (r^2^ < 0.001) to ensure IV independence (28). Since only a single IV was available, the Wald ratio method was applied to estimate the MR effect, with statistical significance set at *p* < 0.05. To evaluate whether GWAS and eQTL signals originate from the same causal variant, Bayesian colocalization analysis was conducted using the “coloc” R package (29). This approach calculates posterior probabilities (PP) for five hypotheses (30), with PP.H4 indicating the likelihood that GWAS and eQTL signals share a causal variant, interpreted as follows:PP.H4 > 0.75: Strong evidence supporting colocalization of GWAS and eQTL signals; PP.H4 between 0.5 and 0.75: Moderate colocalization evidence, suggesting potentially shared causal variants, requiring further validation;PP.H4 < 0.5: Weak colocalization evidence, indicating GWAS and eQTL signals likely originate from different causal variants.

### GeneMANIA analysis

To further explore the biological functions and potential molecular mechanisms of the identified candidate genes, we performed functional enrichment analysis using GeneMANIA^5^ (31). GeneMANIA integrates gene co-expression, protein-protein interactions (PPI), co-regulation, pathway information, and functional similarity data to construct a gene interaction network and predict functional modules and regulatory relationships. This study primarily focused on the potential roles of candidate genes in ALD-related biological processes, including lipid metabolism, inflammation regulation, and hepatic fibrosis.

### Drug enrichment analysis and molecular docking

This study utilized Enrichr^6^ and DGIdb (Drug-Gene Interaction Database^7^, (32, 33) to perform drug enrichment analysis, aiming to identify potential therapeutic drugs targeting C4orf36, HSD17B13, and AFF1 for ALD. Candidate genes were input into the databases, and significantly enriched drug-gene interactions (FDR < 0.05) were selected. Potential drug candidates were further screened from DrugBank, ChEMBL, and DrugMatrix databases (34), and their interactions with target genes were validated. For molecular docking analysis, three-dimensional protein structures were retrieved from the AlphaFold Protein Structure Database^8^ and were selected based on a predicted Local Distance Difference Test (pLDDT) confidence score > 70 to ensure structural reliability. The molecular structures of candidate drugs were downloaded from PubChem or ChEMBL in SDF format (35). Docking simulations were performed using AutoDock Vina (36), with the following key parameters: the grid box size was set to 30 × 30 × 30 Å centered on the predicted or canonical binding pocket, and the exhaustiveness parameter was set to eight. Docking site selection was based on either the known ligand binding pocket or the most probable surface cavity predicted by AutoSite. Compounds with binding free energy ≤ −6 kcal/mol were considered to have high affinity and potential therapeutic relevance.)

## Results

### Cross-tissue and single-tissue TWAS analyses

In the cross-tissue TWAS analysis, 232 genes were identified (*p* < 0.05, Supplementary Table 1), with 12 remaining significant after FDR correction (FDR < 0.05, Table 1). In the single-tissue TWAS analysis, 50 genes showed significance in at least one tissue (FDR < 0.05, Supplementary Table 2). Ultimately, seven candidate genes met the stringent selection criteria (FDR < 0.05) in both cross-tissue and single-tissue analyses, including: Six protein-coding genes: HSD17B13, AFF1, KLHL8, PTPN13, C4orf36, and HSD17B11; One non-coding gene: RP11-476C8.2 (Supplementary Table 3).

**TABLE 1:** The significant genes for alcohol-associated liver disease (ALD) risk in cross-tissue unified test for molecular signatures (UTMOST) analysis.

Gene	*P*-value	ID	CHR	FDR
RP11-476C8.2	2.84E-12	ENSG00000248196.1	4	1.06E-08
HSD17B13	2.11E-07	ENSG00000170509.11	4	2.61E-04
MEPE	2.49E-07	ENSG00000152595.16	4	2.61E-04
AFF1	2.79E-07	ENSG00000172493.20	4	2.61E-04
KLHL8	4.34E-07	ENSG00000145332.13	4	3.25E-04
PTPN13	5.75E-07	ENSG00000163629.12	4	3.58E-04
DMP1	2.46E-06	ENSG00000152592.13	4	1.31E-03
C4orf36	3.00E-05	ENSG00000163633.10	4	1.40E-02
RP11-367N14.2	4.08E-05	ENSG00000232648.3	4	1.69E-02
IBSP	5.00E-05	ENSG00000029559.6	4	1.87E-02
HSD17B11	7.40E-05	ENSG00000198189.10	4	2.52E-02
FAM13A	0.000134923	ENSG00000138640.14	4	4.21E-02

### Conditional and joint analysis

To verify the independence of identified associations and minimize false positives due to linkage disequilibrium (LD), COJO analysis was performed on the seven candidate genes located on chromosome 4 within their respective tissues (Supplementary Figure 1 and Supplementary Table 4). In Colon_Sigmoid, conditioning on KLHL8 significantly weakened the C4orf36 TWAS signal (Supplementary Figure 1A). In Heart_Left_Ventricle, conditioning on HSD17B11 led to a marked reduction in the AFF1 TWAS signal (Supplementary Figure 1B). In Pituitary, conditioning on C4orf36 notably diminished the HSD17B13 TWAS signal (Supplementary Figure 1C). In Skin_Sun_Exposed_Lower_Leg, conditioning on both HSD17B13 and KLHL8 significantly reduced the C4orf36 TWAS signal (Supplementary Figure 1D). In Spleen, conditioning on C4orf36 substantially weakened the AFF1 TWAS signal (Supplementary Figure 1E). In Brain_Anterior_Cingulate_Cortex_BA24, conditioning on HSD17B11, KLHL8, and PTPN13 led to a significant reduction in the HSD17B13 TWAS signal (Supplementary Figure 1F).

### Gene analysis using MAGMA

**FIGURE 2:**
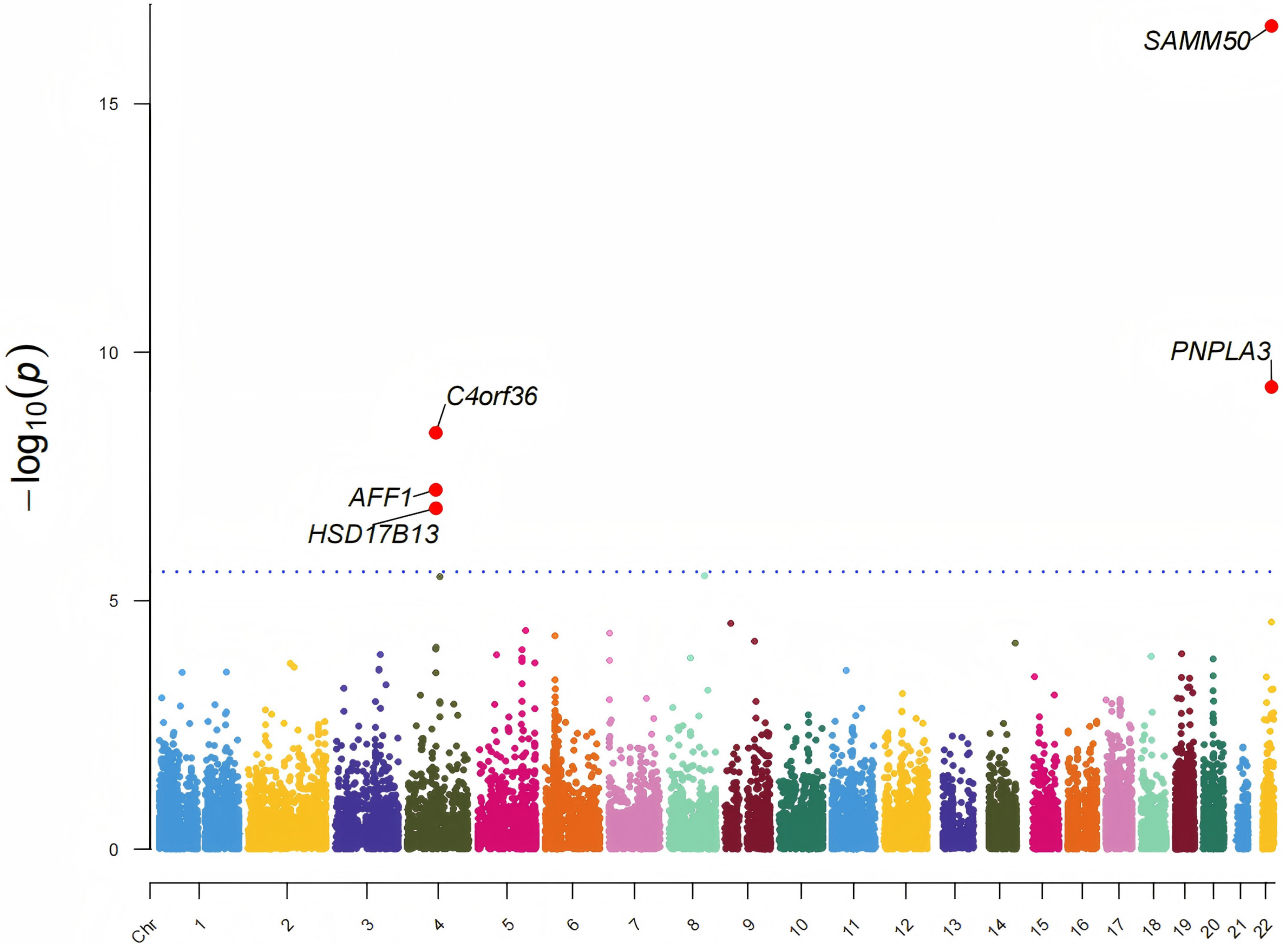
Manhattan plot displaying the gene-level association results from the multi-marker analysis of GenoMic annotation (MAGMA) analysis for alcohol-associated liver disease (ALD). Each point represents a gene, plotted according to its chromosomal position (X-axis) and association strength [–log_10_(P) value, Y-axis]. The top significantly associated genes are highlighted and labeled. The blue dotted horizontal line indicates the false discovery rate (FDR) significance threshold of 0.05. Genes above this line are considered significantly associated with ALD after FDR correction.

**FIGURE 3:**
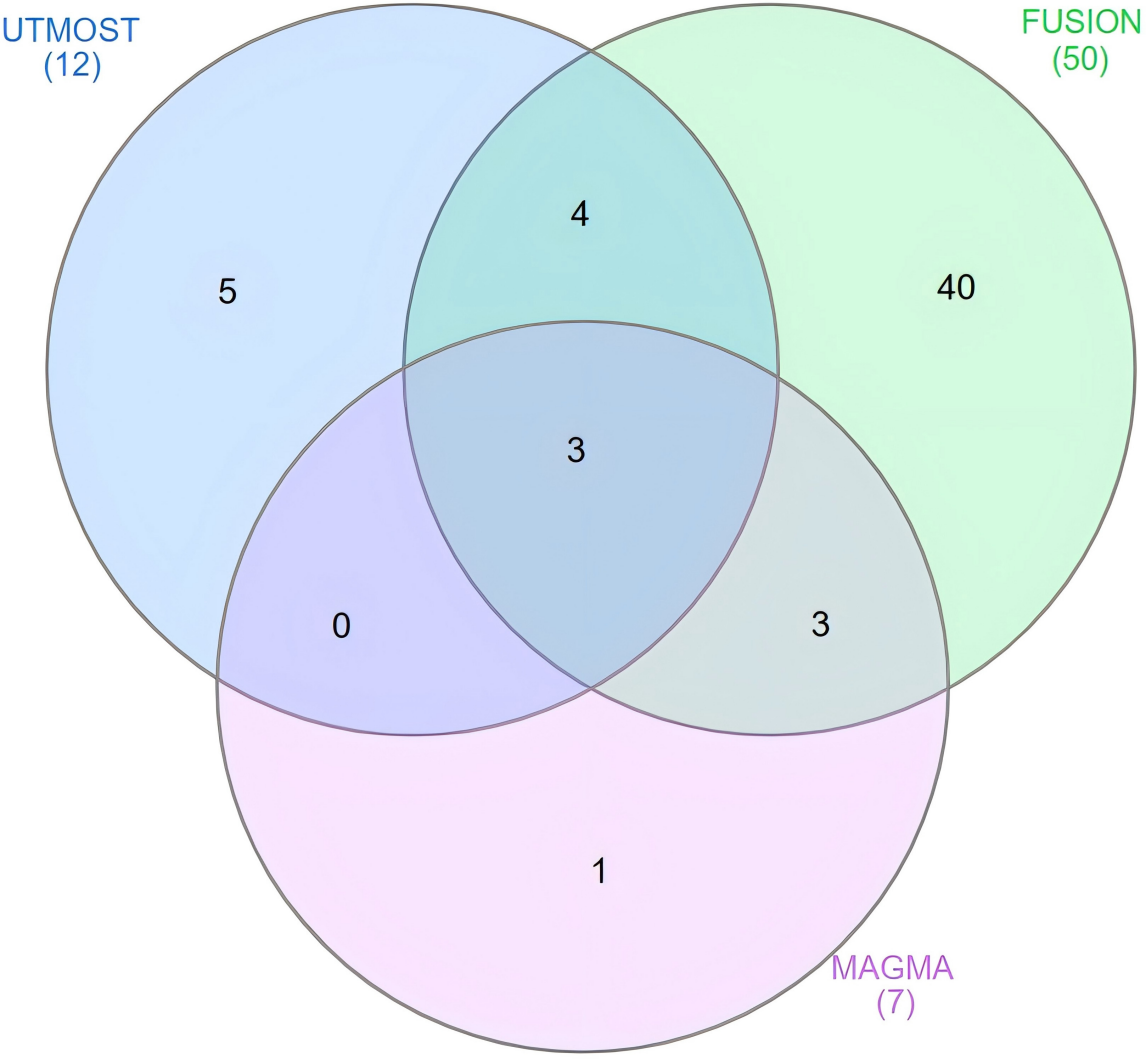
Venn diagram. Multi-marker analysis of GenoMic annotation (MAGMA) identified seven significant genes associated with alcohol-associated liver disease (ALD), functional summary-based imputation (FUSION) identified 50, and unified test for molecular signatures (UTMOST) cross-tissue analysis identified 12, of which 3 were common.

Multi-marker analysis of GenoMic annotation gene-level analysis identified seven genes significantly associated with alcohol-associated liver disease (ALD) (FDR < 0.05, Figure 2 and Supplementary Table 5). To strengthen the reliability of our findings, we integrated significant genes from UTMOST cross-tissue analysis, FUSION, and MAGMA. This comprehensive approach highlighted three key candidate genes: HSD17B13, AFF1, and C4orf36 (Figure 3).

### MR and colocalization analyses

**FIGURE 4:**
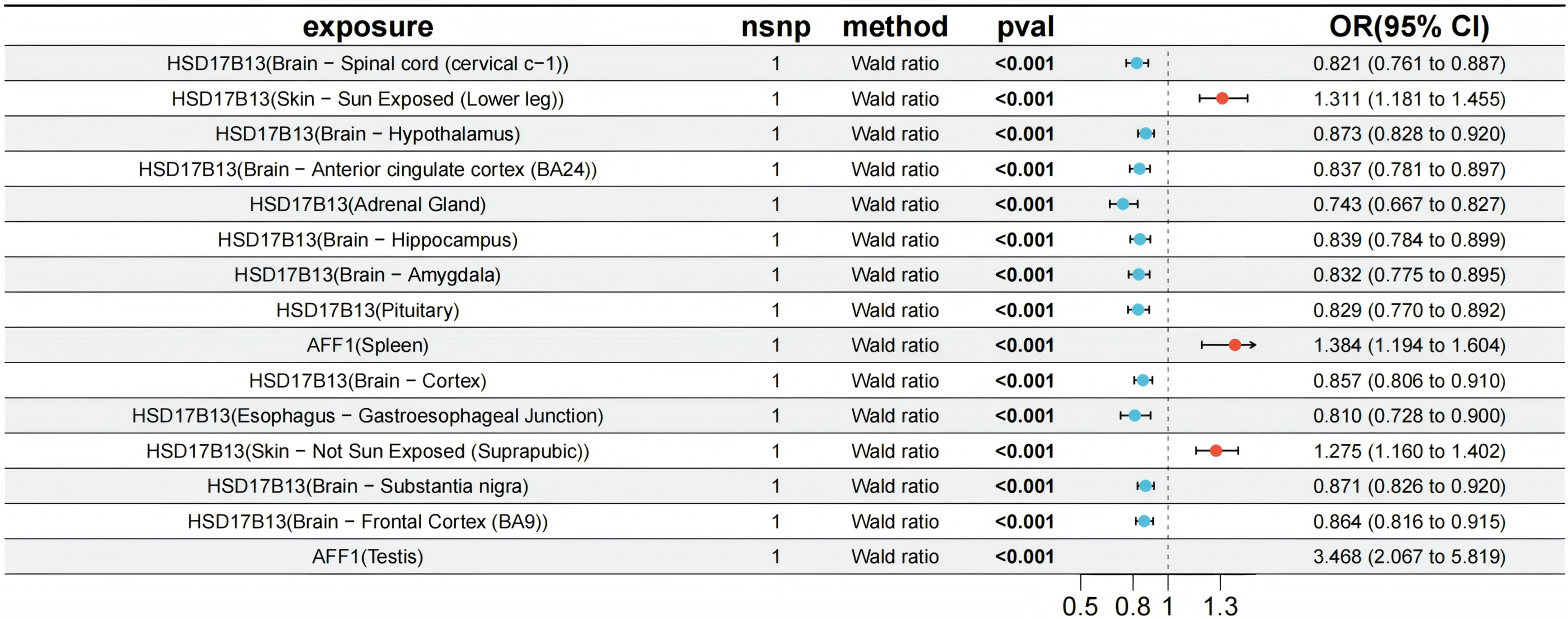
The Mendelian randomization (MR) results confirmed the causal associations between two candidate genes and migraine.

HSD17B13 is located in the chromosome 4q22.1 region. FUSION analysis indicated that its predicted expression levels in multiple tissues (Adrenal_Gland, Artery_ Coronary,Brain_Amygdala,Brain_Anterior_cingulate_cortex_BA- 24,Brain_Cortex,Brain_Frontal_Cortex_BA9,Brain_Hippocampus, Brain_Hypothalamus, Brain _ Spinal _ cord _ cervical_ c-1 , Brain _ Substantia_nigra,Esophagus_Gastroesophageal_Junction,Pituitary, Skin_Not_Sun_Exposed_Suprapubic, and Skin_Sun_Exposed_ Lower_leg) were significantly associated with the risk of alcohol-associated liver disease (ALD) (FDR < 0.05). Mendelian randomization (MR) analysis was conducted in 13 of these tissues, providing further evidence of a potential causal relationship between HSD17B13 and ALD (*p* < 0.05). The odds ratios (ORs) with 95% confidence intervals (CIs) for each tissue were as follows:1.311 (1.181, 1.455), 1.275 (1.160, 1.402), 0.873 (0.828, 0.920), 0.871 (0.826, 0.920), 0.864 (0.816, 0.915), 0.857 (0.806, 0.910), 0.839 (0.784, 0.899), 0.837 (0.781, 0.897), 0.832 (0.775, 0.895), 0.829 (0.770, 0.892), 0.821 (0.761, 0.887), 0.810 (0.728, 0.900), and 0.743 (0.667, 0.827) (Figure 4 and Supplementary Table 6).

However, in Artery_Coronary, no suitable instrumental variables (IVs) were identified, preventing effective MR analysis. Colocalization analysis revealed that HSD17B13 showed minimal evidence of colocalization with ALD in the Esophagus_Gastroesophageal_Junction tissue (PP.H4 = 0.008), suggesting that gene expression and disease risk in this tissue do not share the same genetic variants (Supplementary Figure 2A and Supplementary Table 7). In contrast, PP.H4 values of 0.533 and 0.549 in Skin_Not_Sun_Exposed (Suprapubic) and Skin_Sun_Exposed (Lower leg) tissues, respectively, indicate moderate evidence of colocalization, suggesting the possibility of shared genetic variation (Supplementary Figures 2B, C and Supplementary Table 7).

**FIGURE 5:**
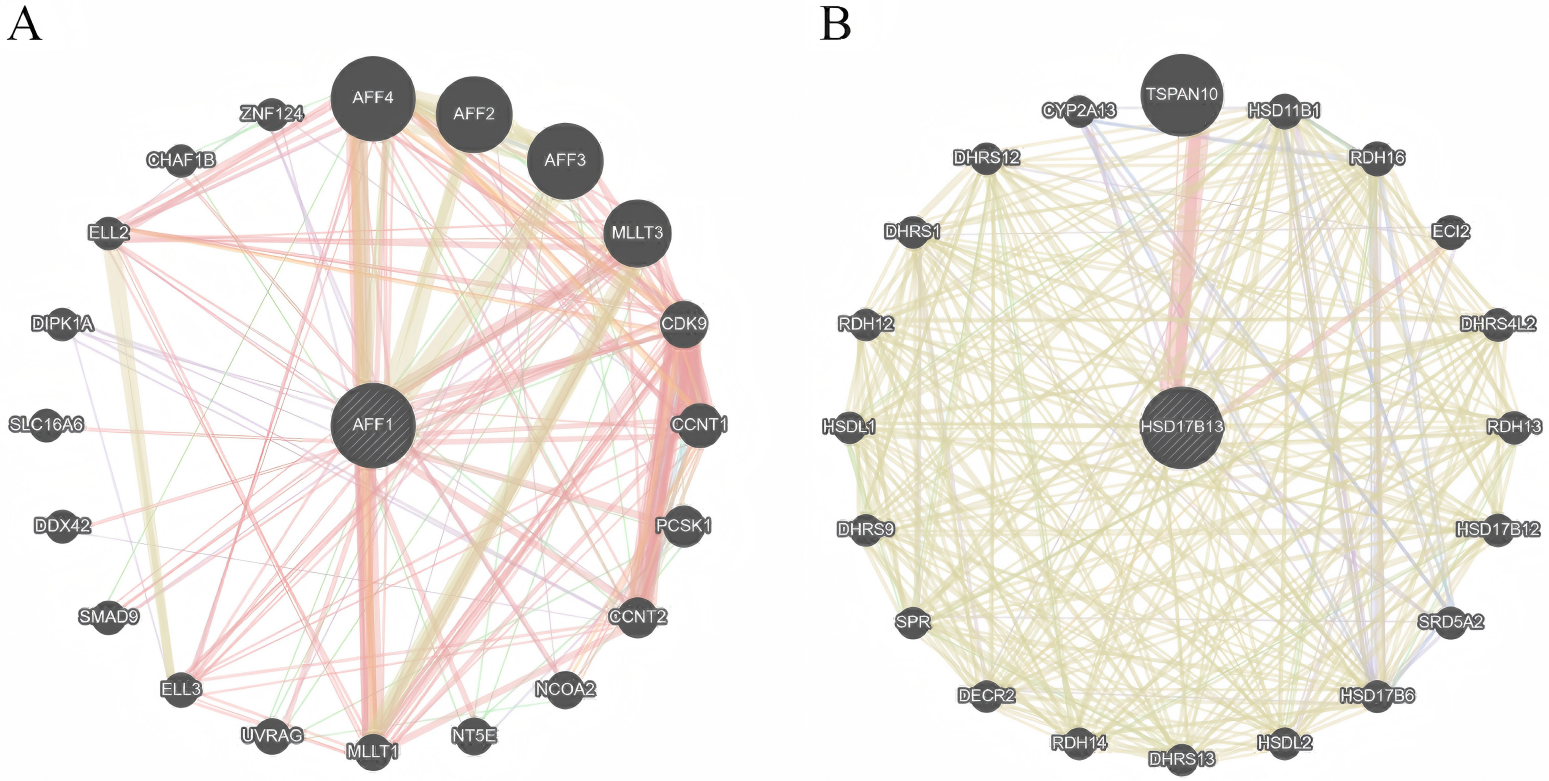
GeneMANIA gene network. **(A)** AFF1 as the core, and **(B)** HSD17B13 as the core.

The AFF1 gene is located in the chromosome 4q21.3 region. FUSION analysis revealed that its predicted expression levels in the spleen, testis, left ventricle (Heart_Left_Ventricle), and frontal cortex (Brain_Frontal_Cortex_BA9) were significantly associated with the risk of alcohol-associated liver disease (ALD) (FDR < 0.05). Mendelian randomization (MR) analysis further provided evidence of a potential causal relationship between AFF1 gene expression and ALD (*p* < 0.05). Specifically, in the testis and spleen, the odds ratios (ORs) with 95% confidence intervals (CIs) were 3.468 (2.067, 5.819) and 1.384 (1.194, 1.604), respectively (Figure 5). However, it should be noted that the colocalization analysis revealed only weak evidence for a shared causal variant (PP.H4 values in both tissues < 0.25) (Supplementary Figures 2D, E and Supplementary Table 7). These results indicate that, despite MR suggesting a possible causal relationship, there is limited support from colocalization, and further studies are needed to clarify the biological mechanism.

Notably, the C4orf36 gene, also located in the chromosome 4q21.3 region, is currently uncharacterized and encodes a protein of unknown function, necessitating further investigation to determine its role in ALD. Due to the limited availability of GWAS data, no suitable instrumental variables (IVs) were identified, precluding reliable Mendelian randomization and colocalization analyses.

### GeneMANIA analysis

The putative gene interaction network centered on HSD17B13 is illustrated in Figure 5A, with the most significantly enriched pathways including steroid metabolism, lipid metabolism, and redox processes (Supplementary Table 8). Similarly, the AFF1-centered gene interaction network (Figure 5B) is predominantly associated with transcriptional regulation, RNA polymerase II-mediated transcriptional elongation, and chromatin modification (Supplementary Table 9). Currently, no functional enrichment data for C4orf36 are available in existing databases. However, its directly associated genes include AC093827.5, HSD3B2, PTGER3, KCND1, and BCO2, which are involved in steroid metabolism, ion channel regulation, and cellular signaling. Given the limited research on C4orf36, its precise biological functions remain to be elucidated through further experimental validation.

### Drug enrichment analysis and molecular docking analysis

**FIGURE 6:**
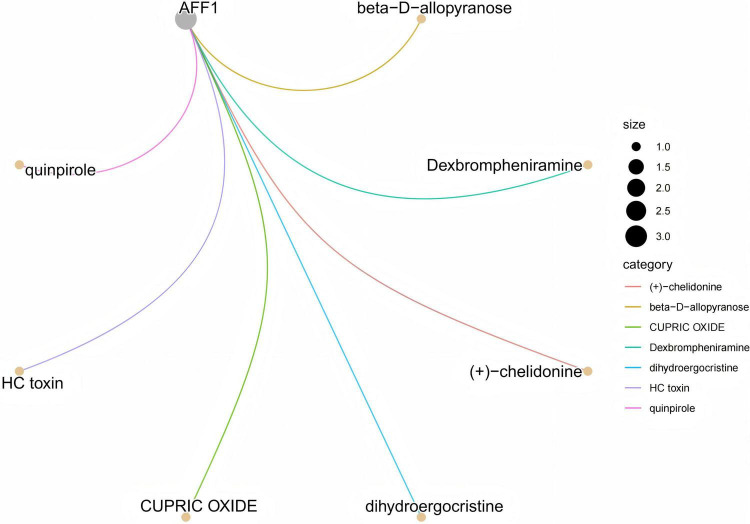
Gene-drug interaction network for AFF1.

Drug enrichment analysis identified three ALD (alcohol-associated liver disease) susceptibility genes—AFF1, C4orf36, and HSD17B13—as potential targets for therapeutic intervention. However, only compounds associated with AFF1 exhibited statistically significant enrichment, whereas no significant results were obtained for C4orf36 and HSD17B13. Therefore, this report primarily focuses on the drug enrichment analysis of AFF1 and its potential biological implications. A total of seven compounds were identified as significantly associated with AFF1, among which beta-D-allopyranose, Dexbrompheniramine, and (+)-Chelidonine exhibited the highest enrichment significance (p.adjust = 0.03785) (Supplementary Table 10 and Figure 6). These compounds are involved in immune regulation, oxidative stress, and nervous system functions, suggesting that AFF1 may play a crucial role in the pathogenesis of ALD. Future studies should further investigate the effects of these compounds on AFF1 and ALD-related pathways to explore their therapeutic potential.

To further evaluate the binding affinity of these candidate compounds to AFF1, molecular docking analysis was conducted on the top three compounds (beta-D-allopyranose, Dexbrompheniramine, and (+)-Chelidonine, with *p* < 0.04), examining their binding modes and molecular interactions with AFF1. The molecular docking results confirmed that these compounds can potentially bind to AFF1 and modulate its function. Among them, (+)-Chelidonine exhibited the lowest Vina docking score (−36.5 kcal/mol), indicating the most stable binding interaction with AFF1 (Supplementary Figures 3, 4).

## Discussion

In this study, we systematically identified candidate susceptibility genes for alcohol-associated liver disease (ALD) by integrating large-scale GWAS datasets and GTEx v8 eQTL data using a cross-tissue transcriptome-wide association study (TWAS) with the UTMOST method. Validation was performed using FUSION analysis, MAGMA analysis, Mendelian randomization (MR), and colocalization analyses. Ultimately, we identified three potential ALD susceptibility genes: HSD17B13, AFF1, and C4orf36.

Drug enrichment analysis further identified several potential therapeutic compounds targeting AFF1, with beta-D-allopyranose, Dexbrompheniramine, and (+)-chelidonine showing the highest enrichment significance (p.adjust = 0.03785). Molecular docking supported the binding potential of these drugs to AFF1, with (+)-chelidonine demonstrating the most favorable docking score. While these results highlight AFF1 as a promising therapeutic target, it is important to note that molecular docking predictions are hypothesis-generating; actual drug efficacy and safety require rigorous *in vitro* and *in vivo* experimental validation. These findings offer a preliminary framework for the translational development of AFF1-targeted therapies in ALD.

From a clinical translation perspective, AFF1-targeted drug development remains in the preclinical stage. Currently, there are not clinically approved small-molecule inhibitors or biologics specifically targeting AFF1. The most relevant advances have come from oncology, where efforts have focused on disrupting the MLL-AF4 fusion protein or modulating AFF1-driven transcriptional complexes in acute lymphoblastic leukemia. These studies underscore the feasibility of targeting AFF1 but also highlight key challenges, including achieving target specificity, minimizing off-target effects, and ensuring efficient delivery to hepatic and immune cell types relevant to ALD. For example, (+)-chelidonine, identified in our docking screen as a top candidate, has demonstrated anti-inflammatory and anti-proliferative activities in previous cell-based models, but its affinity for AFF1 and efficacy in liver disease contexts are untested. Furthermore, the physiological role of AFF1 in immune regulation and transcriptional control suggests that global inhibition could disrupt normal hematopoiesis or immune homeostasis, raising safety concerns for clinical translation.

Recent GWAS studies have revealed several genetic loci associated with ALD risk, such as PNPLA3, TM6SF2, MBOAT7, MARCI, and HNF1A (6, 9, 10). Moreover, HSD17B13 has been increasingly recognized as associated with ALD (7, 37–41). In this study, we not only reconfirmed the role of HSD17B13 but also identified AFF1 and C4orf36 as novel susceptibility genes previously unreported in the context of ALD.

HSD17B13 is a lipid-droplet-associated protein widely expressed in liver tissues. Previous research has extensively demonstrated that loss-of-function variants of HSD17B13 confer protection against both non-alcoholic fatty liver disease (NAFLD) and ALD (7, 38). For instance, Abul-Husn et al. (7) found in a large clinical cohort that the loss-of-function variant rs72613567 significantly reduced the risk of liver disease progression to cirrhosis (7). In our study, cross-tissue TWAS and MR analyses provided further evidence supporting a causal relationship between HSD17B13 expression and ALD risk. MR analyses revealed significant associations between HSD17B13 expression levels and ALD risk in 13 different tissues (e.g., Brain_Hippocampus, Brain_Hypothalamus, Skin_Not_Sun_Exposed_Suprapubic). Colocalization analysis suggested moderate evidence of shared genetic variants between HSD17B13 and ALD in skin tissues (PP.H4 = 0.533–0.549), whereas no significant colocalization was observed in Esophagus-Gastroesophageal Junction tissue (PP.H4 = 0.008). These results indicate tissue-specific roles of HSD17B13 in ALD. GeneMANIA analysis further implicated steroid metabolism, lipid metabolism, and redox processes as potential pathways through which HSD17B13 could modulate lipid accumulation and inflammation-fibrosis progression in hepatocytes, thereby influencing ALD pathogenesis. Although loss-of-function variants of HSD17B13 are associated with reduced risk of NAFLD and ALD, its therapeutic potential remains controversial. Vilar-Gomez et al. (39) noted that current evidence is mainly from genetic associations and animal studies, lacking direct clinical intervention data (39). Ma et al. (40) highlighted that its mechanisms and efficacy vary by disease type and individual differences, indicating heterogeneity in treatment response (40). Liu et al. (41) cautioned that the long-term safety and effectiveness of HSD17B13 inhibition are still uncertain, with potential differences in benefit among populations requiring further investigation. Thus, while promising, HSD17B13’s clinical application requires rigorous large-scale validation.

AFF1 (AF4/FMR2 Family Member 1, also known as AF4) is a gene located on chromosome 4q21.3. AFF1 encodes a nuclear protein that functions primarily as a transcriptional regulator. It is a key member of the AF4/FMR2 (AFF) family, which is involved in regulating gene expression and RNA polymerase II transcription elongation. AFF1 is best known for its role in hematological malignancies, especially acute lymphoblastic leukemia (ALL), where chromosomal translocations involving AFF1 (such as MLL-AF4 fusion) lead to leukemogenesis (42, 43). Beyond leukemia, recent transcriptomic studies and single-cell analyses have shown that AFF1 is expressed in various tissues, including immune and hepatic tissues, and may participate in immune regulation, cellular signaling, and inflammation. Functionally, AFF1 has been implicated in modulating transcriptional elongation, mRNA processing, and cellular responses to external stimuli, suggesting broader roles in health and disease beyond hematologic cancers (44–46). Given its functional involvement in immune regulation and inflammation—two central processes in ALD pathogenesis—AFF1 may contribute to hepatic injury and immune-mediated disease progression, providing a plausible mechanistic link between its expression and ALD susceptibility. Our study identified AFF1 as significantly associated with ALD for the first time, supported by multiple analytical approaches. FUSION analysis showed significant associations between predicted AFF1 expression levels in multiple tissues (spleen, testis, left ventricle, and frontal cortex) and ALD risk. MR analysis further supported causal relationships for AFF1 expression in testis (OR = 3.468, 95% CI: 2.067–5.819) and spleen (OR = 1.384, 95% CI: 1.194–1.604). Nevertheless, colocalization analyses indicated weak evidence for shared genetic signals in these tissues (PP.H4 = 0.054 for testis; PP.H4 = 0.212 for spleen), suggesting potential horizontal pleiotropy or other confounding factors. Further functional validation (e.g., CRISPR-Cas9 gene editing, single-cell RNA sequencing) is required to clarify AFF1’s precise role in ALD pathology. GeneMANIA analysis also suggested AFF1 involvement in immune regulation, transcriptional control, and cellular signaling pathways, processes closely linked to immune dysregulation and chronic inflammation in ALD (47). Coupled with drug enrichment and molecular docking results, these findings support AFF1 is a promising candidate for further investigation, offering novel insights for ALD genetic mechanisms and targeted therapies.

Notably, the C4orf36 gene emerged as a newly discovered genetic risk locus with very limited prior research and unclear biological function. Although our cross-tissue TWAS analysis preliminarily associated C4orf36 expression with ALD risk, reliable MR and colocalization analyses were not possible due to the lack of suitable instrumental variables (IVs). Future functional studies are required to elucidate C4orf36’s biological significance and mechanisms in liver disease.

A significant methodological strength of our study is the application of cross-tissue TWAS (UTMOST), which effectively integrates multi-tissue eQTL data, enhancing prediction accuracy and stability of identified genes (12, 13). Validation through joint analyses (FUSION, MAGMA, MR, colocalization) further ensured reliability of our findings. Additionally, this is the first study to perform drug enrichment and molecular docking analysis targeting ALD susceptibility genes, providing critical clues for exploring potential therapeutic targets.

Nevertheless, several limitations merit consideration. First, the GWAS data used were predominantly from European populations, limiting generalizability to other ethnic groups such as Asian or African populations. Large-scale, multi-center validations in diverse populations are warranted. Second, despite comprehensive bioinformatics validations, direct experimental evidence (cellular experiments or animal models) is lacking, necessitating further validation of our findings. Third, clinical and biochemical parameters such as ALT, AST, and fibrosis scores were not available in the current summary-level datasets, precluding analysis of associations between genetic variation and disease-relevant clinical phenotypes. This limits the clinical interpretability of our results and highlights the need for future studies incorporating richer phenotypic data. Lastly, considering ALD complexity, future studies should integrate additional environmental factors (e.g., drinking patterns, dietary habits) to more deeply explore gene-environment interactions.

Despite these limitations, our integrative multi-omics approach identified novel genetic susceptibility genes and proposed new avenues for targeted therapy in ALD. Importantly, these findings serve as testable hypotheses, providing a foundation for future experimental and functional validation to further elucidate ALD pathogenesis and treatment.

## Data Availability

Publicly available datasets were analyzed in this study. This data can be found here: the genome-wide association study (GWAS) data for alcohol-associated liver disease (ALD) were sourced from the FinnGen R12 dataset (https://r12.finngen.fi/pheno/K11_ALCOLIV). Gene expression and expression quantitative trait loci (eQTL) data were obtained from the GTEx V8 database, which is freely accessible at https://ftp.ebi.ac.uk/pub/databases/spot/eQTL/ imported/GTEx_V8.

## Footnotes

1
https://www.finngen.fi/en

2
https://github.com/Joker-Jerome/UTMOST

3
http://gusevlab.org/projects/fusion/

4
https://ctg.cncr.nl/software/magma

5
https://genemania.org/

6
https://maayanlab.cloud/Enrichr/

7
https://www.dgidb.org/

8
https://www.alphafold.ebi.ac.uk/
