# Supplementary material for: Genetic insights into alcohol-associated liver disease: integrative transcriptome-wide analysis identifies novel susceptibility genes
Source: Front Med (Lausanne). 2025 Jul 31;12:1623367. doi: 10.3389/fmed.2025.1623367 (PMC12351384; doi:10.3389/fmed.2025.1623367)
Supplement: Supplementary file 1 [file Data_Sheet_1.docx]

**
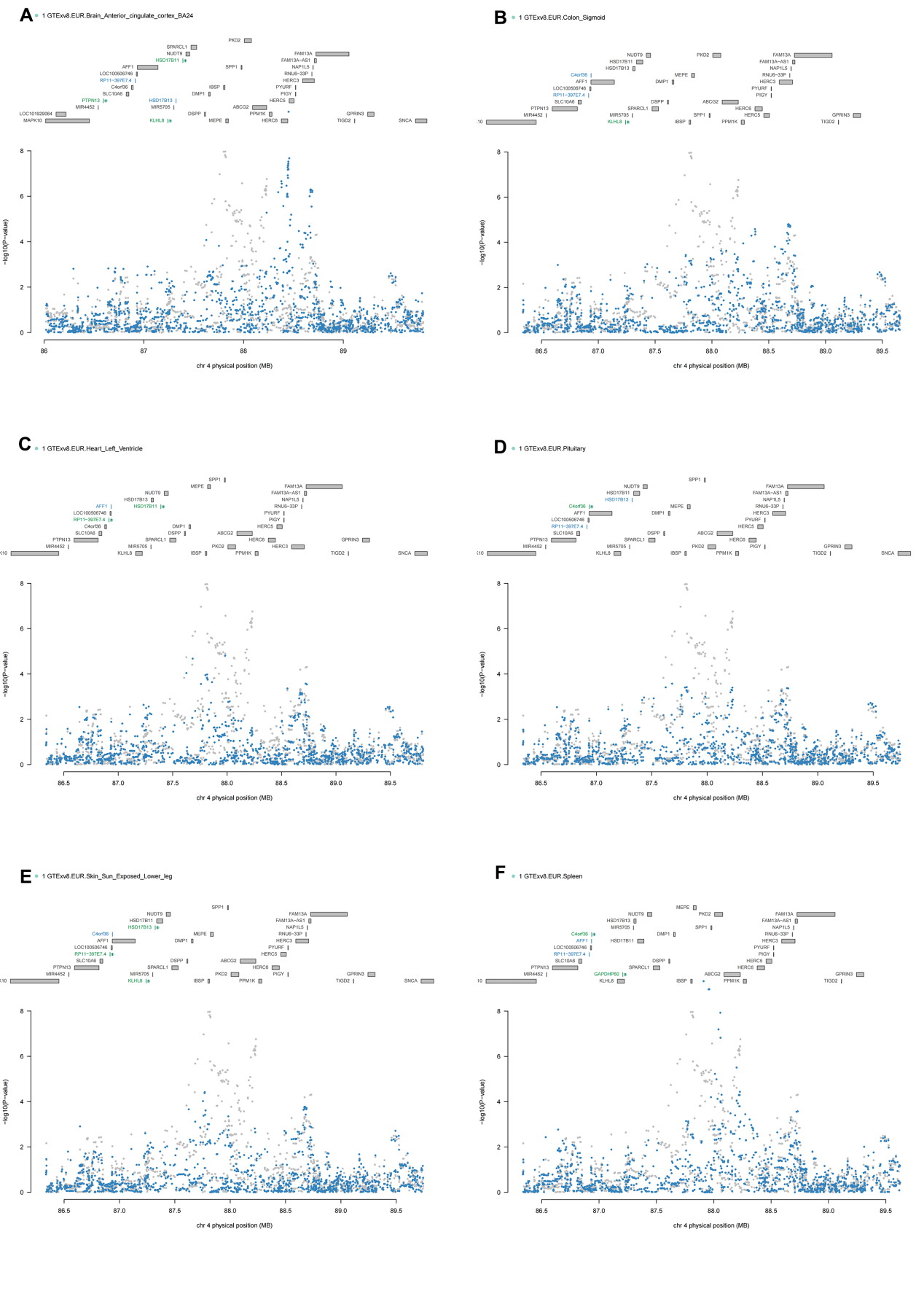
**

**Figure S1. Regional association of TWAS hits and conditional analysis in six representative tissues.**

For each locus, the upper panel displays all genes within the region. Marginally significant TWAS genes (P < 0.05 in single-gene analysis) are shown in blue, while jointly significant genes (remaining significant after conditional and joint analysis, FDR < 0.05) are shown in green. The lower panel presents regional Manhattan plots of GWAS association results: grey points indicate GWAS p-values before conditioning, and blue points show GWAS p-values after conditioning on the predicted expression of jointly significant (green) genes. The attenuation of GWAS signals after conditioning indicates that the TWAS-identified genes explain a substantial proportion of the GWAS association at these loci.


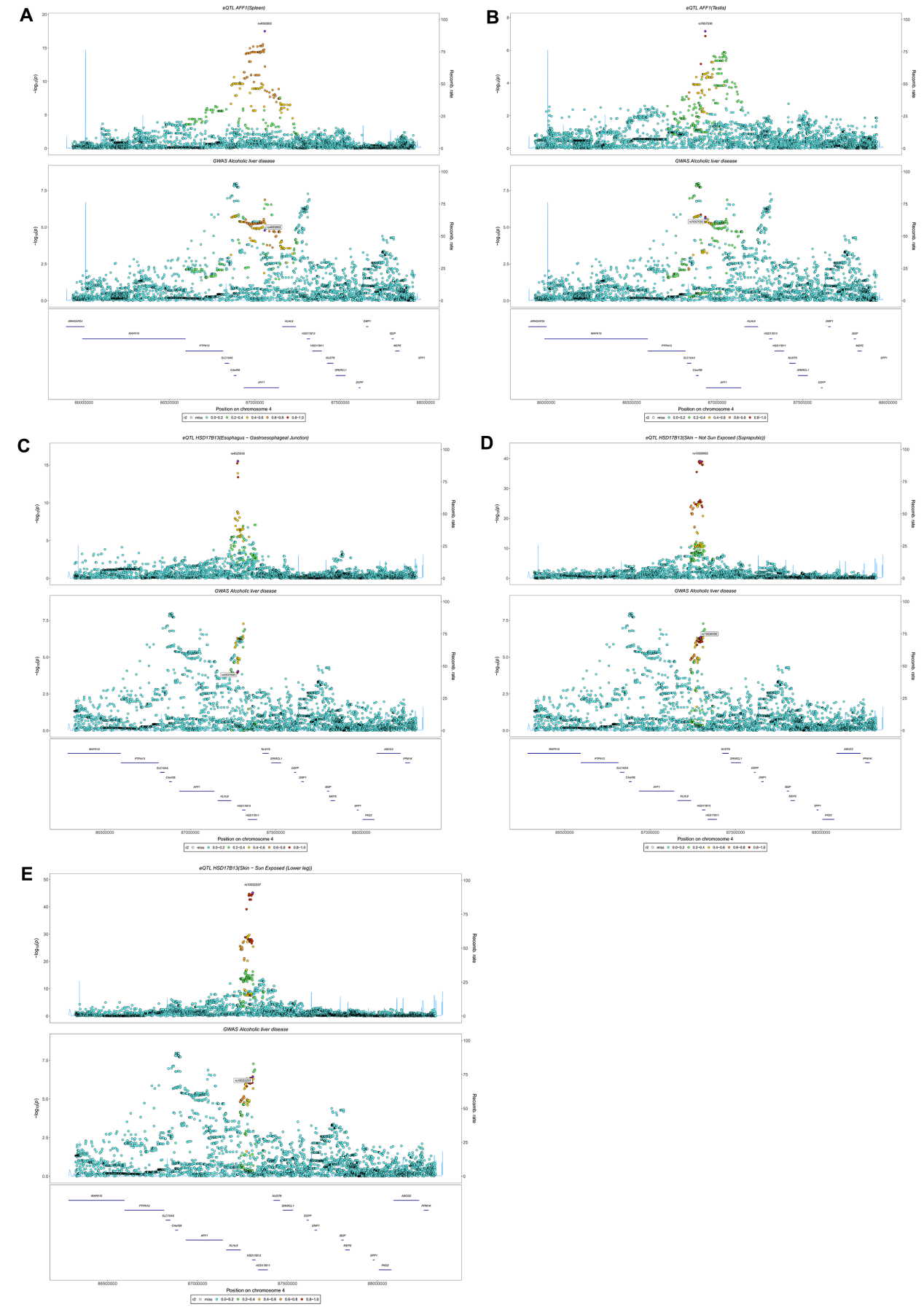


**Figure S2.**The results of colocalization analysis between candidate genes and alcohol-associated liver disease (ALD). The SNP rs17022564 exhibited the lowest cumulative sum of ALD GWAS and REV1 eQTL p values both in Whole_Blood (A) and Cells_Cultured_fibroblasts (B). The SNP rs738248 exhibited the lowest cumulative sum of ALD GWAS and SREBF2 eQTL p values both in Skin_Sun_Exposed_Lower_leg (C) and Testis (D)

**
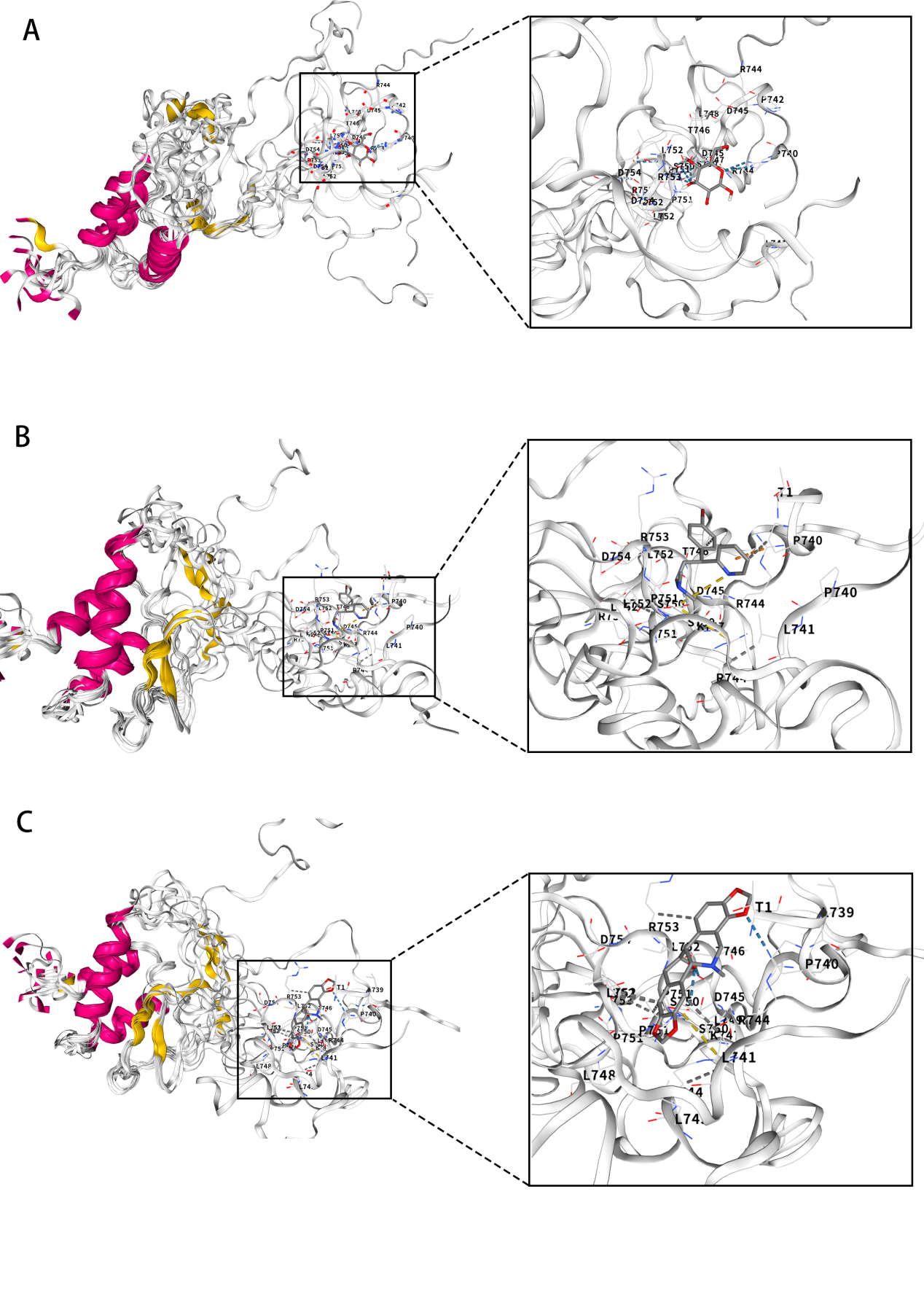
Figure S3.**Molecular docking analysis of drug compounds with AFF1.(A) Docking of beta-D-allopyranose with AFF1.(B) Docking of Dexbrompheniramine with AFF1.(C) Docking of (+)-Chelidonine with AFF1.





**Figure S4. Pocket-level molecular docking outcomes of beta-D-allopyranose, Dexbrompheniramine, and (+)-chelidonine with AFF1.**

(A) Docking results of beta-D-allopyranose with AFF1, showing the best binding affinity at pocket C2 (Vina score = –25.0 kcal/mol).

(B) Docking results of Dexbrompheniramine with AFF1, with the strongest interaction observed at pocket C2 (Vina score = –28.6 kcal/mol).

(C) Docking results of (+)-chelidonine with AFF1, exhibiting the lowest Vina score (–36.5 kcal/mol) at pocket C2, indicating the most stable binding.
